# Evaluation of the benefits of adapted physical activity in children and adolescents with osteogenesis imperfecta: the MOVE-OI trial

**DOI:** 10.1186/s13023-025-03678-4

**Published:** 2025-04-12

**Authors:** Hayssam Al Arab, Sacha Flammier, Morgane Espitalier, Justine Bacchetta, Marine Fouillet-Desjonqueres

**Affiliations:** 1 Department of Pediatric Nephrology, Rheumatology and Dermatology, Hôpital Femme Mère Enfant, Bron, France; 2 Reference Center for Rare Calcium and Phosphate Diseases, OSCAR Network, European Network for Rare Bone Diseases BOND, Bron, France; 3 INSERM Research Unit 1033 LYOS, Lyon 1 University, Lyon, France; 4 Competence Center for Constitutional Bone Diseases, OSCAR Network, Bron, France

**Keywords:** Osteogenesis imperfecta (OI), Bone fragility, Adapted physical activity (APA), Pediatric children, 6-min walk test, Prospective trial, Bone density, Respiratory function, Endurance, Physical capacity

## Abstract

**Background:**

Osteogenesis Imperfecta (OI) is a rare genetic disorder characterized by bone fragility and susceptibility to fractures. No curative treatment currently exists, and limited data are available on the effects of adapted physical activity (APA). This study evaluates the impact of APA on bone health, physical function, respiratory function, and quality of life in pediatric children with OI.

**Methods:**

The MOVE-OI trial (NCT 04119388) is a prospective single-center study assessing the impact of a 12-month individualized APA program. Inclusion criteria included confirmed OI pathogenic variant, ages 6–18 years. Baseline (M0) and end-point (M12) assessments included clinical, radiological, and respiratory evaluations. The primary outcome was an improvement in the 6-min walk test (6MWT) distance. A non-parametric paired-test was performed for analysis.

**Results:**

Thirty participants (16 males, median age 10.5 years) completed the program. A 17% increase in the 6MWT distance (*p* = 0.0007) was observed, with an average improvement of 98 m. No significant bone density or respiratory function changes were detected. Fracture incidence decreased (from 40 to 20%), and quality-of-life improvements were noted in participants with high baseline difficulty scores.

**Conclusion:**

APA improves endurance and physical capacity in children with OI. Multidisciplinary care and further research are needed to enhance long-term outcomes.

## Introduction

Osteogenesis Imperfecta (OI) is a rare genetic disorder (prevalence ~ 1/15,000) characterized by reduced bone mass, fragility, and fracture susceptibility of varying severity [1]. Most cases (~ 90%) are attributed to pathogenic variants in type 1 collagen genes (COL1A1/COL1A2). Diagnosis today typically includes clinical and genetic assessments [2]. OI presents with skeletal symptoms and extra skeletal manifestations, including muscle weakness, bluish sclera, dentinogenesis imperfecta, and fatigue [3].

Current treatments primarily focus on reducing fracture incidence and improving quality of life in individuals with OI. Despite its potential importance, the role of physical activity (PA) in influencing bone health and managing OI remains insufficiently explored [4]. The World Health Organization (WHO) defines PA as skeletal muscle-induced movement increasing energy expenditure. Adapted physical activity (APA) is a multidisciplinary field that focuses on tailoring PA, sport, and exercise to meet the needs of individuals with disabilities or special needs, promoting inclusion, accessibility, and well-being [5].

PA plays a crucial role in reducing premature mortality by mitigating the risk of various chronic diseases, such as cardiovascular disease, cancer, and type 2 diabetes. A systematic review and dose–response meta-analysis demonstrated that sedentary behavior is associated with increased risks of these conditions, while increased PA provides protective benefits [6].

Despite established PA benefits for bone health and quality of life, data specific to pediatric OI populations are sparse. Reduced aerobic capacity and muscle atrophy in OI necessitate targeted interventions [7].

In these children, the genetic variant and its associated consequences result in reduced aerobic capacity, muscle atrophy, impaired rehabilitation, and hindered development of musculoskeletal, cardiac, and pulmonary tissues [8]. Consequently, multidisciplinary management and continuity of care are essential, necessitating close collaboration between families and healthcare professionals. Together, they aim to guide and support these children in achieving maximum autonomy [9].

Several articles recommend a regular exercise program for patients with OI, but do not say what it consists of or explain what it is based on [5, 10].

We decided to work on APA in this peculiar disorder, keeping in mind this daily paradox: OI is a bone-weakening disorder (which leads to reduced autonomy) that may oblige us to overprotect affected children, whereas, in contrast, these recommendations encourage all patients to go beyond their illness and practice APA. Thus, one might ask, what approaches to APA accepted in the literature might be considered, and what would be the benefits of these various programs for children and young people with OI.

This study investigates the impact of APA on physical endurance, respiratory function, and quality of life in children with OI. The goal is to demonstrate that a 12-month individualized APA program can improve physical function, particularly endurance, as measured by the 6MWT, in children and adolescents with OI. It is hypothesized that this APA program will not only improve the 6MWT distance but also have beneficial effects on bone health, respiratory function, and quality of life, potentially reducing fracture incidence and enhancing the physical capacities of participants.

## Methods

### Study design and participants

The MOVE-OI study is a prospective trial conducted at Hospices Civils de Lyon (November 2019–October 2021). Participants (n = 30) were enrolled from the Constitutional Bone Diseases Competence Center, out of a total of approximately 100 children. The study was introduced at a child’ day meeting and received feedback from the Osteogenesis Imperfecta Association (OIA) and the initiative “Move toi avec ta MOVE,” which promotes PA for individuals with OI. “Move toi avec ta MOVE” is a play on words in French that combines the idea of movement (“Move toi,” meaning “Get moving”) with the name of the study, “MOVE”.

Inclusion required confirmed OI pathogenic variants (Table 1), ages 6–18, and informed consent. The exclusion criterion was an orthopedic contraindication to APA (n = 0).

**Table 1:** Descriptive table of the study population

	N = 30
Gender (M)	16
Median age ± σ (years; min–max)	10.5 ± 3.42 (6.2–16.6)
Gene (number of children)	
*COL1A1*	20
*COL1A2*	7
*IFITM5*	1
*THEM38B*	1
Not detected	1
Disease type (number of children), based on genetic variant	
Moderate familial	25
Severe familial	1
Moderate de Novo	3
Severe de Novo	1
Bisphosphonates before M0 (number of children)	17
Fractures (number of children)	
Within the 12 months before inclusion	12
Between M0-M12	6
Median weight Z-score	0.37
Range of weight Z-scores	−2.41 – 3.40
Median height Z-score	− 0.70
Range of height Z-scores	−6.94 – 3.85
Engagement in sport activities (number of children)	24
Outside school activities (number of children)	19

### Procedures

Baseline and follow-up assessments (M0 and M12) encompassed clinical, radiological, respiratory evaluations and APA sessions. Radiological assessments included Dual X-ray Absorptiometry (DXA) to measure bone density, while respiratory evaluations involved the Tiffeneau ratio, forced expiratory volume in one second (FEV1), and functional vital capacity (FVC). The APA session incorporated the 6-min walk test (6MWT), exercise instructions, and demonstrations of exercises to be performed at home. These exercises, conducted biweekly for 30 min, focused on improving cardiovascular health, coordination, and muscle strengthening. The exercise instructions for home practice were provided by the APA instructor.

Contact with each child was maintained through the same instructor, who communicated with the parents via telephone at 1, 3, and 9 months. Regular interviews were conducted to ensure follow-up, assess progress, and adapt PA participation.

Participants were given two questionnaires (APA assessment and PedsQL (quality of life in pediatrics), and provided with a booklet, which they completed at each visit (M0-M12).

At the 6-month mark (M6), participants underwent a clinical check-up, consultations with the referring physician and dietician, and another APA session, along with the completion of the two questionnaires.

### Outcomes

Primary Outcome: Improvement in 6MWT distance from M0 to M12.

Secondary Outcomes: Bone density (spine and femoral neck), quality of life, fracture incidence, and respiratory function stability.

### Statistical analysis

Non-parametric paired tests analyzed before/after differences. Results are median (IQR). PRISM software was used for analysis.

The analysis includes Z-scores calculated using WHO Growth Standards: placeholder LMS (L: Lambda, M: Median, S: Sigma) values. For intermediate ages not directly covered by the LMS dataset, linear interpolation was applied.

## Results

### Participants’ characteristics

Baseline characteristics are displayed in Table 1. The study included 30 participants, consisting of 16 boys and 14 girls, with a median age of 10.5 ± 3.42 (6.2–16.6) years. Most participants (83%, n = 25) had a moderate familial form of OI (based on genetic variant), whilst 10% (n = 3) had a moderate de novo form. Regarding the severe form, 3.3% (n = 1) of participants had a familial form and 3.3% (n = 1) had a de novo form. Over half of the participants (n = 17) were receiving bisphosphonate treatment prior to inclusion in the program and continued treatment throughout the study. During the year preceding the APA program (M 12), 40% of participants (n = 12) experienced fractures, whereas only 20% (n = 6) experienced fractures between M0 and M12 (p = 0.7). Median height Z-score was− 0.70, with a range from− 6.94 to 3.85, while the median weight Z-score was 0.37, ranging from− 2.41 to 3.40. Of the 30 participants, 80% engaged in school sports (football (soccer), basketball, handball, volleyball, athletics, gymnastics, and swimming), and approximately 63% participated in sports activities outside of school (Sports Clubs: Football, Tennis, swimming, Gymnastics). Engagement in PA, both inside and outside of school, was assessed through questionnaires. Participants were asked whether they engage in school-based physical activities and, if not, to specify reasons such as pain-related exemptions, medical contraindications, teacher restrictions, or other factors. Additionally, they were asked if they participate in sports outside of school, with follow-up questions regarding their current involvement and the types of sports practiced.

Most children were highly motivated to participate in the program, with a mean motivation score of 9.9/10. All participants had normal vitamin D and calcium intakes, as well as adequate daily caloric intake.

### 6-min walk test (6MWT)

**Fig. 1:**
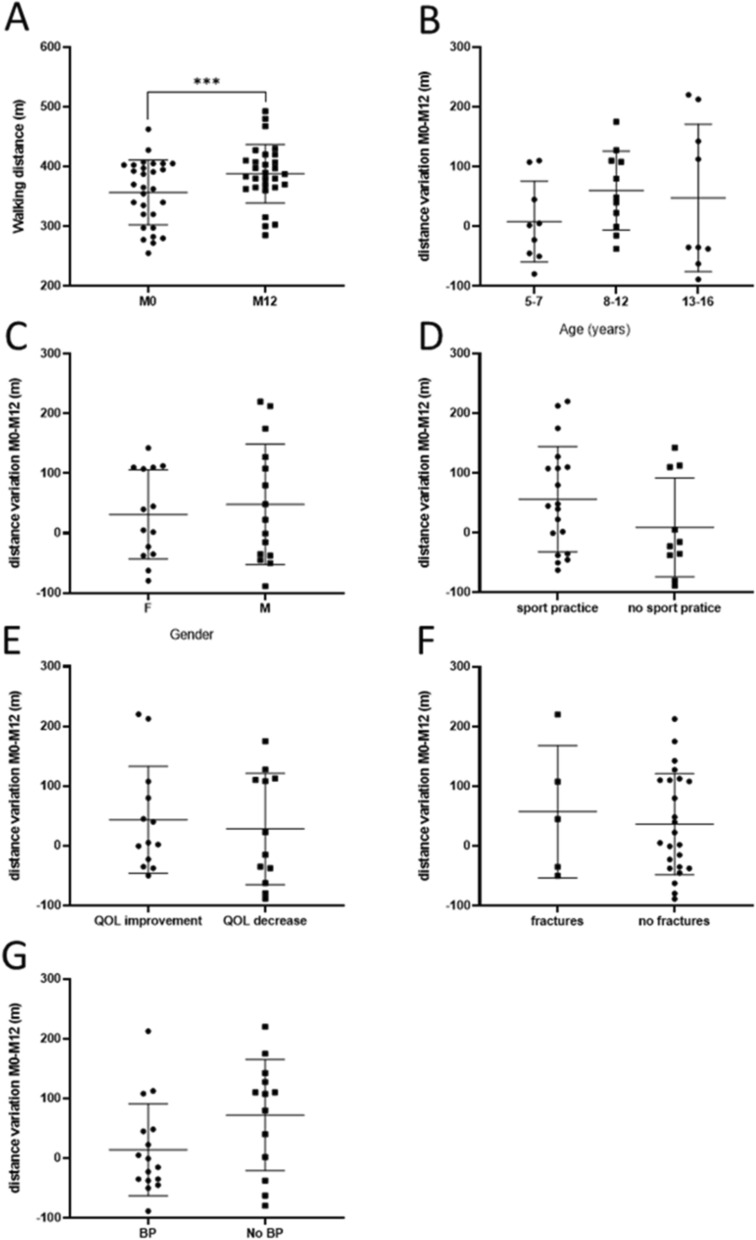
Six-minute Walk test (6MWT). **A**: Distance covered (m) at M0 and M12, **B**: Delta 6MWT versus age, **C**: Relationship between the Delta 6MWT and the gender, **D**: Relationship between the Delta 6MWT and the sport activity, **E**: Relationship between the Delta 6MWT and the quality of life, **F**: Relationship between the Delta 6MWT and the fractures, **G**: Relationship between the Delta 6MWT and the treatment by Bisphosphonates (BP)

There was a significant 17% increase in the distance covered during the 6-min walk test (6MWT) in children with OI after a 12-month APA program, with an increase of 98 m in average in over half of the children (57%) (Fig. 1A). There were no significant differences in the 6MWT results based on age, sex, sport practice, quality of life, bisphosphonate treatment, or fracture episodes (Fig. 1B–G).

### Quality of Life

Quality of life was assessed using a difficulty score, a lower score indicating better quality of life. There was no significant reduction in the difficulty score overall. However, children who initially experienced the most difficulties at baseline (M0) demonstrated a decrease in their score, indicating an improvement in their quality of life.

**Fig. 2:**
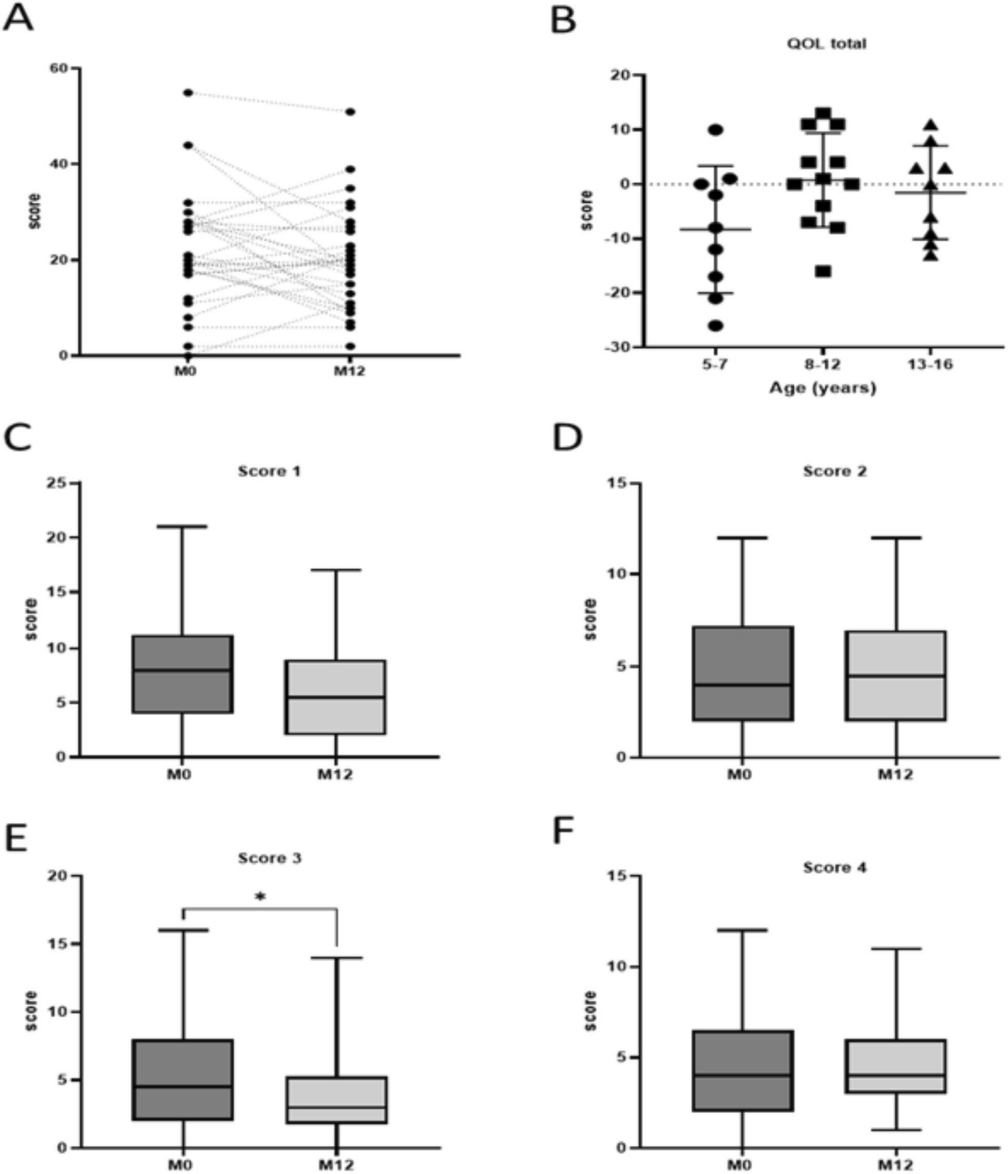
Effect of APA program on participants' quality of life (QOL). **A**: QOL score between M0 and M12, **B**: score difference between M0 and M12 depending on age (5–7; 8–12; 13–16 years-old), **C-F:** difficulty score subgroups (**C**: Score 1: my health and activities; **D**: Score 2: my emotions, **E:** score 3: relationship with others; **F** score 4: my studies)

The questionnaire used in the assessment comprised various scores. A notable improvement was observed in Score 3, which is likely associated with social interaction and the development of social skills, cohesion, and a sense of belonging to the team, particularly in the presence of the APA teacher (Fig. 2A–F).

### Bone mineral density

The Z scores for bone mineral density remained stable or showed a positive trend at the spine level in all children except two. Conversely, a stability or decline was observed at the femoral neck level in all children except two.

**Fig. 3:**
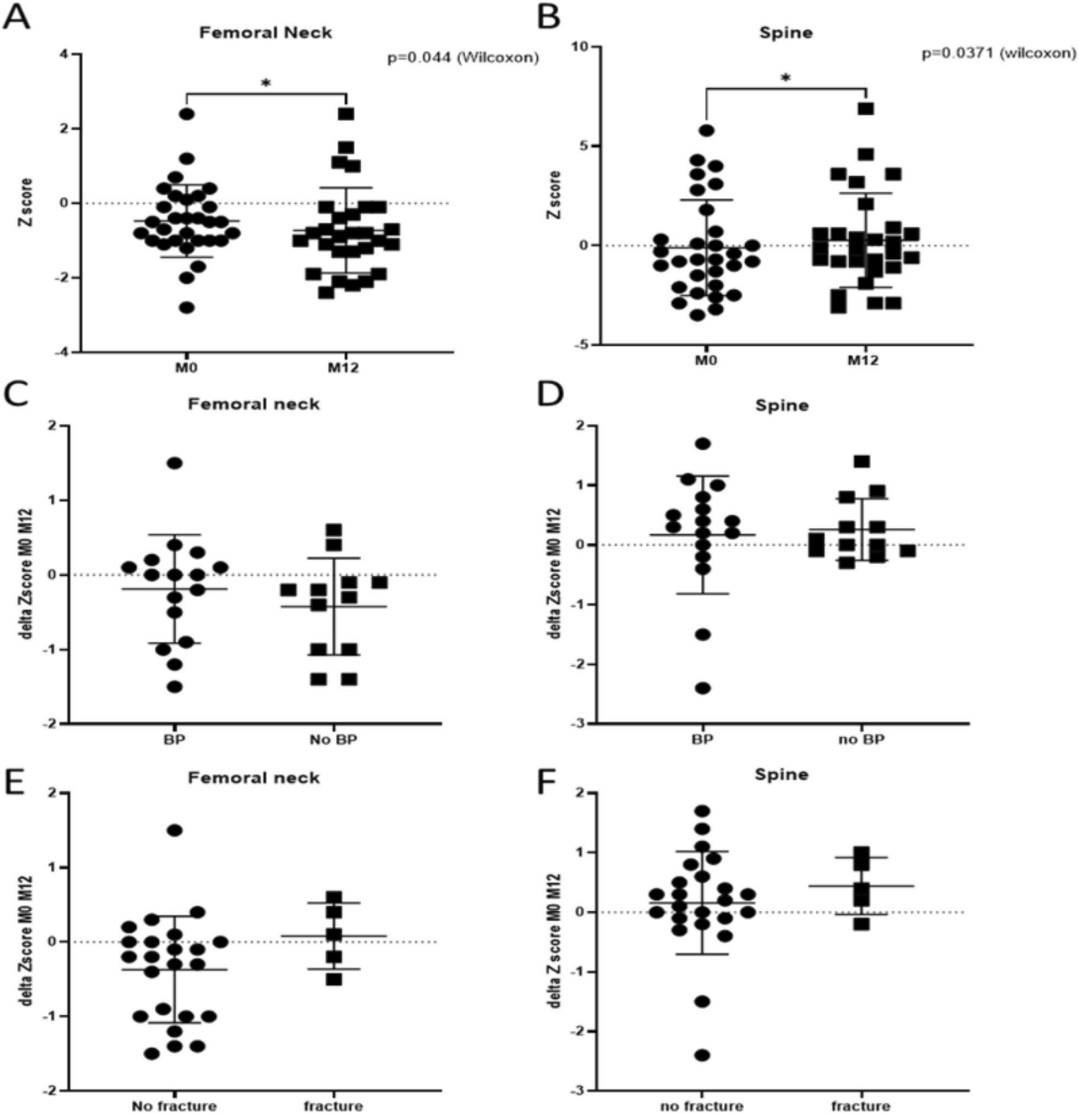
Femoral neck and spine bone density. **A**: Effect of APA on bone density of the femoral neck; **B**: Effect of APA on bone density of the spine, **C**: Relationship between the femoral neck density and the bisphosphonates treatment (BP), **D**: Relationship between the spine density and the bisphosphonates treatment (BP); **E**: Relationship between the femoral neck density and the fractures; **F:** Relationship between the spine density and the fractures

Despite these observations, there was no discernible effect of fracture episodes on femoral neck or spine bone density (Fig. 3C–F).

### Respiratory function test

**Fig. 4:**
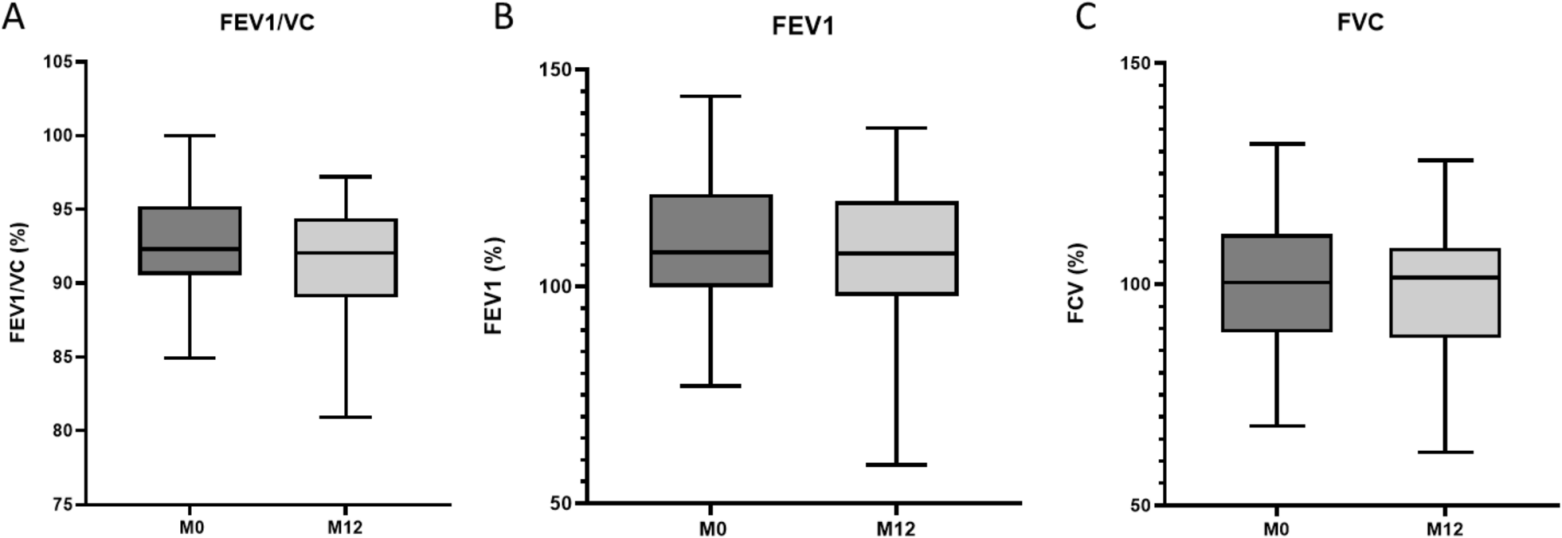
Effect of APA program on respiratory function testing. **A**: Tiffeneau FEV1/VC; **B**: FEV1: forced expiratory volume in one second; **C**: FVC: functional vital capacity

Throughout the duration of the APA program, respiratory function parameters showed no significant changes (Fig. 4).

## Discussion

This study contributes to the limited body of research examining the effects of APA on endurance and physical capacity in children with OI. Previous research has shown that typically developing children can experience an annual improvement of 3–12% in walking distance regardless of specific physical activities [11]. Notably, Hoyer-Kuhn et al. [12] demonstrated a 25% improvement in physical capacity among children with OI following a year-long APA regimen. Similar improvements in PA benefits for children with OI, such as muscle strength, fatigue, motor function, and walking distance, have been reported in other studies. For example, Van Brussel et al. observed a 12% improvement in muscle strength after a 12-week graduated exercise program [13], while another study reported enhanced mobility based on GMFM tests and the 1-min walk test after a whole-body vibration training program [12]. Our findings revealed a 17% improvement in physical capacity, as measured by the 6MWT, following a 12-month APA program in children with OI. Comparatively, Hoyer-Kuhn et al. reported a more substantial 25% improvement in physical capacity after a year-long APA regimen conducted in a specialized rehabilitation center. Several factors may account for these differences. The Hoyer-Kuhn et al. study involved a structured, center-based program with higher supervision and likely greater adherence to the exercise regimen. In contrast, our approach relied on parent-managed home-based sessions, which, while accessible, may have introduced variability in program intensity and consistency. Additionally, differences in patient demographics, baseline physical capacity, or disorder severity could have contributed to the observed variation. It is also important to note that the COVID-19 pandemic disrupted PA patterns and limited access to resources, potentially influencing outcomes in our study.

With respect to bone mineral density (BMD), Hoyer-Kuhn et al. found significant improvements in total body BMD without the head, whereas our study observed stability or minimal changes. This may be attributable to differences in the exercise modalities used. While our program focused on general PA, it included limited targeted weight-bearing or impact-loading exercises, which are more effective in stimulating bone remodeling. Furthermore, the home-based nature of our intervention may have influenced adherence and the intensity of exercises performed.

Despite these differences, our study provides valuable insights into the feasibility and benefits of a home-based APA program, which offers a more accessible and sustainable approach for families of children with OI. Improvements in physical endurance and reductions in fracture incidence demonstrate the potential of APA to enhance functional outcomes and quality of life. These findings emphasize the importance of integrating tailored APA programs into the routine care of children with OI, particularly in settings where access to specialized rehabilitation centers is limited.

In addition to physical capacity, PA has a recognized benefit for bone health, particularly by enhancing bone mineral density in younger individuals. It exerts pressure on the skeleton, promoting bone strength and health [14]. Research on osteoporosis has long established that physical inactivity is a risk factor for fractures and osteoporosis, while movement and muscle mass gain are protective [15]. Similar findings have been observed in children, where bone mineral density can be enhanced during the growth period [16]. Our study also revealed increased spine bone density after a 12-month APA regimen, with no corresponding rise in fracture risk. The stability of the femoral neck observed in our study may be influenced by the effects of bisphosphonate treatment, which is commonly prescribed children with OI to improve bone strength. However, further research would be needed to clarify the precise interaction between bisphosphonates and the impact of PA on bone health in this population.

The APA sessions in our study incorporated passive stretching, low-impact, weight-bearing exercises to enhance muscle strength and bone health. The program included aerobic exercises for cardiovascular fitness along with activities aimed at improving coordination, balance, and overall physical function. As emphasized in the consensus statement by Mueller et al., tailored rehabilitation programs combining these elements are essential to improving physical function and quality of life in children and adolescents with OI [16].

These exercises are consistent with guidelines recommending low to moderate aerobic activity, such as swimming, for children with OI, particularly those aged up to 12, with type I or IV OI. Intense exercise sessions should be avoided in young children, while older children should refrain from contact sports and avoid sudden rotational movements [4, 17, 18 and 19].

Despite the benefits, children with OI face numerous barriers to engaging in PA. Fear of fractures, overprotection by parents, and limited mobility often lead to reduced PA and further immobility, creating a cycle that is difficult to break. Addressing these barriers through personalized therapeutic approaches and psychological support is crucial for improving child engagement in PA [16]. Additionally, weight gain during puberty, coupled with reduced mobility, poses an obesity risk for children with OI. Therefore, weight management strategies and consultations with nutritionists are vital [16]. In particular, children with OI often face significant psychosocial challenges, including potential stigmatization and social isolation, particularly in environments such as school physical education classes. These challenges can adversely affect their quality of life and overall life satisfaction [20]. Furthermore, a consensus statement on physical rehabilitation in children and adolescents with OI recommends a coordinated, multidisciplinary team approach to ensure that children with OI can fulfill their potential by maximizing function, independence, and societal participation [16].

A comparative study involving control or reference groups would have bolstered the credibility of our results. However, given the limited number of children with OI (around 30 in Lyon), the variability in OI severity, and the wide age range (6–17 years), creating comparable groups would have been impractical. The variability in OI severity and fracture history would have compromised the statistical power and validity of the results. Further, matching children based on characteristics such as age, sex, and OI type would have added another layer of complexity and could have hindered meaningful subgroup analyses.

While the duration of a year-long study is necessary to observe potential bone health benefits, child motivation presented a challenge during the COVID-19 lockdowns. Despite these difficulties, the study successfully maintained child engagement, standing in contrast to the general trend of increased sedentary behavior among children during the pandemic. While the 6MWT was an appropriate tool for assessing initial physical capacity, a more detailed exercise test could have provided deeper insights into the participants’ functional capacities.

## Conclusion

In conclusion, this pioneering study provides valuable initial data on the advantages of APA for children with OI. Engagement with the study protocol, led to increased activity and helped families overcome their reservations, encouraging them to incorporate more PA into their daily lives beyond the study. Our findings underscore the significant potential of APA to improve physical capacity, bone health, and overall quality of life in children with OI. These results highlight the critical need for ongoing research to refine and optimize PA programs tailored to this population. Ultimately, integrating APA into routine clinical management is essential for enhancing long-term outcomes and supporting the well-being of children with OI.

## Data Availability

The datasets generated and/or analyzed during the current study are available from the corresponding author on reasonable request.
